# Multivalent Aptamer‐Based Lysosome‐Targeting Chimeras (LYTACs) Platform for Mono‐ or Dual‐Targeted Proteins Degradation on Cell Surface

**DOI:** 10.1002/advs.202308924

**Published:** 2024-02-29

**Authors:** Qiao Duan, Hao‐Ran Jia, Weichang Chen, Chunhong Qin, Kejing Zhang, Fei Jia, Ting Fu, Yong Wei, Mengyang Fan, Qin Wu, Weihong Tan

**Affiliations:** ^1^ Institute of Molecular Medicine (IMM) Renji Hospital Shanghai Jiao Tong University School of Medicine Shanghai Jiao Tong University Shanghai 200120 China; ^2^ Hangzhou Institute of Medicine (HIM) Chinese Academy of Sciences Hangzhou Zhejiang 310022 China; ^3^ Department of General Surgery Xiangya Hospital Central South University Changsha Hunan 410006 China

**Keywords:** aptamer, LYTACs, multivalency, protein degradation

## Abstract

Selective protein degradation platforms have opened novel avenues in therapeutic development and biological inquiry. Antibody‐based lysosome‐targeting chimeras (LYTACs) have emerged as a promising technology that extends the scope of targeted protein degradation to extracellular targets. Aptamers offer an advantageous alternative owing to their potential for modification and manipulation toward a multivalent state. In this study, a chemically engineered platform of multivalent aptamer‐based LYTACs (AptLYTACs) is established for the targeted degradation of either single or dual protein targets. Leveraging the biotin‐streptavidin system as a molecular scaffold, this investigation reveals that trivalently mono‐targeted AptLYTACs demonstrate optimum efficiency in degrading membrane proteins. The development of this multivalent AptLYTACs platform provides a principle of concept for mono‐/dual‐targets degradation, expanding the possibilities of targeted protein degradation.

## Graphical abstract

This study introduces a highly efficient and versatile multivalent aptamer‐based LYTAC platform achieved through the conjugation of multiple aptamers with bpM6P. By harnessing the unique advantages of aptamers, this platform shows potential for the simultaneous degradation of multiple crucial proteins, thus providing a promising strategy for designing potent AptLYTACs with augmented degradation capabilities.

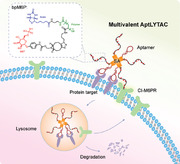

## 1 Introduction

Proteins fundamentally undertake almost every aspect of life's activities in biological systems, and their dysregulation is frequently associated with the pathogenesis of various diseases such as malignancies and neurodegenerative disorders.^[^ ^1^, ^2^ ^]^ Traditional therapeutic strategies rely on small‐molecule inhibitors or antibodies to block the function of specific protein targets.^[^ ^3^, ^4^, ^5^ ^]^ Despite their clinical success, those strategies suffer from drawbacks such as resistance to mutations and the inability to target undruggable proteins.^[^ ^6^ ^]^ Over the past two decades, the emergence of targeted protein degradation technologies, exemplified by proteolysis‐targeting chimeras (PROTACs), has revolutionized our approach to therapeutically relevant proteins.^[^ ^7^, ^8^, ^9^ ^]^ These degraders typically leverage the proteasome‐mediated degradation pathway to catalytically and persistently eliminate proteins of interest (POIs). However, their dependency on the proteasomal machinery imposes limitations on their applicability to cytosolic ligandable domains, thereby precluding the degradation of extracellular and/or cell membrane proteins, which constitute the majority of existing drug targets.^[^ ^9^ ^]^

To overcome this challenge, researchers have been exploring alternative degradation systems based on different mechanisms of action.^[^ ^10^, ^11^ ^]^ One such paradigm is lysosome‐targeting chimeras (LYTACs) reported by Bertozzi's group.^[^ ^12^, ^13^ ^]^ Mechanistically, LYTACs consist of a POI‐targeting antibody and a ligand segment recognized by cell surface lysosome‐shuttling receptors, such as cation‐independent mannose‐6‐phosphate receptor (CI‐M6PR) or asialoglycoprotein receptor, enabling the transport of POIs into lysosomes for degradation. Complementary to PROTACs, LYTACs are specifically designed for secreted and membrane‐associated proteins, thus expanding the range of degradable cellular targets.

**Figure 1:**
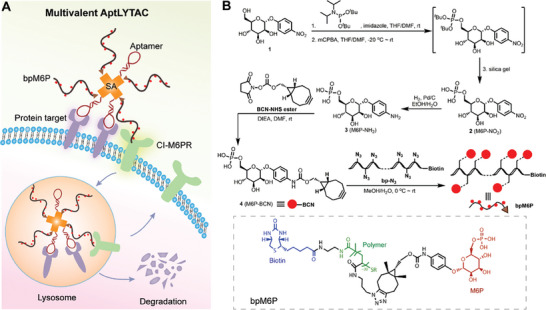
Design of multivalent AptLYTACs. A) Schematic illustration of the multivalent AptLYTACs for targeted degradation of cell surface proteins. B) Synthetic strategy for bpM6P. mCPBA, m‐chloroperbenzoic acid; THF, tetrahydrofuran; rt, room temperature; Pd/C, m‐chloroperbenzoic acid; DIEA, diisopropylethyl amine resin.

However, the current chemical conjugation methods used to construct LYTACs between antibodies and synthetic ligands lack site‐specificity,^[^ ^11^ ^]^ potentially compromising the affinity of antibodies to targets. While precise antibody modifications are technically feasible, they often entail a trade‐off between cost and efficacy.^[^ ^14^ ^]^ Moreover, the large size and potential immunogenicity of antibodies can give rise to issues such as epitope competition and biosafety concerns.^[^ ^15^ ^]^ Recently, many researches demonstrated the feasibility of utilizing aptamers, which are single‐stranded oligonucleotides, to construct LYTAC‐like systems for protein degradation.^[^ ^16^, ^17^, ^18^ ^]^ Aptamers possess high specificity for their targets and have several advantages over antibodies^[^ ^19^ ^]^ including smaller size, cost‐effective synthesis, and precise chemical modifications.^[^ ^20^ ^]^ These advantages make aptamer‐based LYTACs (termed AptLYTACs) a promising tool for protein degradation in clinical applications. Unlike the multivalent nature of antibodies (e.g., bivalent IgG), aptamers are typically monovalent. Although introducing bi/multivalency to aptamers to enhance their specificity and affinity has been widely adopted in the fields of biosensing and bioimaging,^[^ ^21^ ^]^ the relationship between the valency of aptamer binders and the efficacy of AptLYTACs is still unexplored, because previously reported AptLYTACs only contain one aptamer binder in each construct.^[^ ^16^, ^17^, ^18^ ^]^ Moreover, the potential of AptLYTACs to simultaneously degrade more than one protein target has not been uncovered by now. In this study, we report a modular multivalent AptLYTACs platform to explore the relationship between aptamer valency and protein degradation efficiency (**Figure** **1**A). The platform is constructed based on the controlled assembly between biotin and streptavidin (SA), where SA is modified with aptamers at predetermined molar ratios, and biotin is introduced to the poly mannose‐6‐phosphate (bpM6P) polymer targeting cell surface CI‐M6PRs. Our results demonstrate that multivalent AptLYTACs mediate the lysosomal degradation of cell surface proteins more efficiently than their monovalent counterparts, but only within a specific range of SA:aptamer stoichiometric ratios. The versatility of the multivalent AptLYTACs is validated by their consistent effectiveness in degrading different protein targets. Additionally, we evaluate the efficacy of degrading dual protein targets by integrating two distinct aptamers into a single AptLYTAC scaffold. In our proof‐of‐concept experiments, we successfully demonstrate the efficient simultaneous degradation of dual proteins using bi‐specific AptLYTACs. This work highlights the advantage of aptamers in constructing multivalent systems for degrading one or multiple membrane protein targets, potentially paving the way for the design of more potent therapeutic degraders in the future.

## 2 Results and Discussion

### 2.1 Synthesis and Characterization of bpM6P

**Figure 2:**
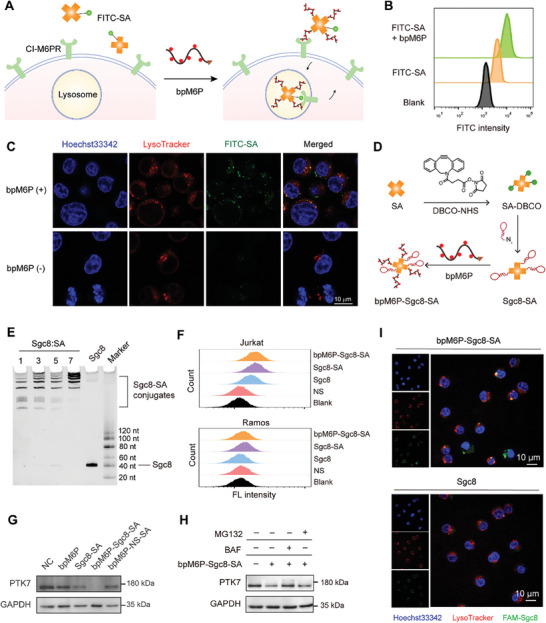
Construction of multivalent AptLYTACs for PTK7‐targeted degradation. A) Scheme illustrating the mechanism of cellular internalization of FITC‐SA mediated by bpM6P. B) Internalization of FITC‐SA in Jurkat cells, as determined by flow cytometry after 1 h treatment with 500 nm FITC‐SA in the presence or absence of 2 µm bpM6P. C) Confocal fluorescence images of Jurkat cells after treatments as described in (B). Before imaging, cells were labeled with LysoTracker Red for 10 min and Hoechst 33342 for 10 min, followed by washing in Dulbecco's phosphate‐buffered saline (DPBS) for three times. Scale bar, 10 µm. D) Synthetic route of Sgc8‐conjugated AptLYTACs. E) Denaturing gel electrophoresis analysis of different Sgc8‐SA conjugates at varying valency ratios of SA:Sgc8 (1:1, 1:3, 1:5, and 1:7). F) Flow cytometry analysis of 100 nm NS, Sgc8, Sgc8‐SA (SA:Sgc8 = 1:3), and bpM6P‐Sgc8‐SA (SA:Sgc8 = 1:3) binding ability in Jurkat and Ramos cells. G) Western blot result of PTK7‐targeted degradation by multivalent AptLYTACs (SA:Sgc8 = 1:3) in Jurkat cells after treatment without (NC) or with Sgc8‐SA (500 nm), bpM6P‐Sgc8‐SA (bpM6P 2 µm, Sgc8‐SA 500 nm), bpM6P‐NS‐SA (bpM6P 2 µm, NS‐SA 500 nm, NS stands for negative sequence), or bpM6P alone (2 µm) for 4 h. H) Western blot result indicating the levels of PTK7 in Jurkat cells pretreated with 100 nm BAF or 1 µm MG132 for 2 h and then treated with 500 nm bpM6P‐Sgc8‐SA for 2 h under the maintenance of corresponding inhibitor. BAF, bafilomycin. I) Confocal fluorescence images of Jurkat cells that were first treated with Sgc8 or bpM6P‐Sgc8‐SA for 4 h and then labeled with LysoTracker Red and Hoechst 33342. Scale bar, 10 µm.

We first synthesized a biotinylated polymeric CI‐M6PR ligand (termed bpM6P) according to the synthetic route shown in Figure 1B. The successful syntheses of different intermediates and the final product bpM6P were verified by ^1^H nuclear magnetic resonance (NMR) and ^31^P NMR (Figures S1–S5, Supporting Information). To assess whether the prepared polymers could be recognized by CI‐M6PRs and mediate cellular internalization, we assembled fluorescein isothiocyanate‐labeled streptavidin (FITC‐SA) protein with bpM6P, which was then used to treat Jurkat cells for 1 h (**Figure** **2**A). Confocal imaging and flow cytometric results collectively revealed a significant internalization of FITC‐SA inside cells, with evident localization in lysosomes (Figure 2B,C), thereby demonstrating the polymer's ability to mediate efficient cellular internalization. In contrast, FITC‐SA alone was not preferred for rapid internalization by Jurkat cells at the same time scale. We next sought to synthesize aptamer‐SA conjugates using a “tag‐and‐modify” approach (Figure 2D). In this method, SA was first reacted with dibenzocyclooctyne‐*N*‐hydroxysuccinimidyl ester (DBCO‐NHS) through the conjugation between NHS ester and the primary amines of SA. Matrix‐assisted laser desorption ionization‐time of flight (MALDI‐TOF) mass spectrometry analysis revealed that approximately 12 DBCO groups were introduced to an SA protein (Figure S6, Supporting Information). Then, aptamers functionalized with azide groups were reacted with the DBCO‐SA via the strain‐promoted alkyne‐azide cycloaddition to generate aptamer‐SA conjugates.

### 2.2 PTK7‐Targeted Degradation by Sgc8‐Conjugated AptLYTACs

As a model, we first utilized Sgc8, a protein tyrosine kinase 7 (PTK7)‐targeting aptamer,^[^ ^22^ ^]^ to generate monovalent and different multivalent aptamer‐SA conjugates by altering the molar ratios of SA:Sgc8 added to reaction systems. Denaturing gel electrophoresis was employed to characterize the aptamer‐SA conjugates after purification. The result revealed an increasing trend of the molecular weight of SA‐Sgc8 conjugates with the increase of Sgc8 valency (Figure 2E), which was determined by gel image‐based quantification analysis (Figures S7 and S8, Supporting Information). Additionally, the assembly of bpM6P with Sgc8‐SA was optimized and characterized by native gel electrophoresis, as shown in Figure S9, Supporting Information. Next, we aimed to evaluate the protein degradation efficiency of AptLYTACs that specifically target PTK7. Initially, we tested their binding specificity through flow cytometric analysis in Jurkat (PTK7+) and Ramos (PTK7−) cell lines, and the results proved that attaching Sgc8 onto the AptLYTAC system does not interfere with aptamer specificity (Figure 2F). Excitingly, western blot result showed that AptLYTACs induced a substantial reduction of PTK7 protein levels in Jurkat cells, while Sgc8‐SA, bpM6P alone, or AptLYTAC with a scrambled nucleic acid sequence (bpM6P‐NS‐SA) displayed negligible PTK7 degradation effect (Figure 2G). These results validate the design rationale of AptLYTACs for targeted PTK7 degradation. Confocal imaging revealed that Sgc8 aptamers were mainly translocated from the plasma membrane to lysosomes upon AptLYTACs treatment, indicating a lysosome‐mediated degradation mechanism (Figure 2I). Moreover, the addition of bafilomycin, a V‐ATPase inhibitor that hinders lysosomal acidification,^[^ ^23^ ^]^ rather than MG132, a proteasome inhibitor,^[^ ^24^ ^]^ effectively restored PTK7 levels in AptLYTACs‐treated cells (Figure 2H), confirming the lysosome‐mediated degradation mechanism.

**Figure 3:**
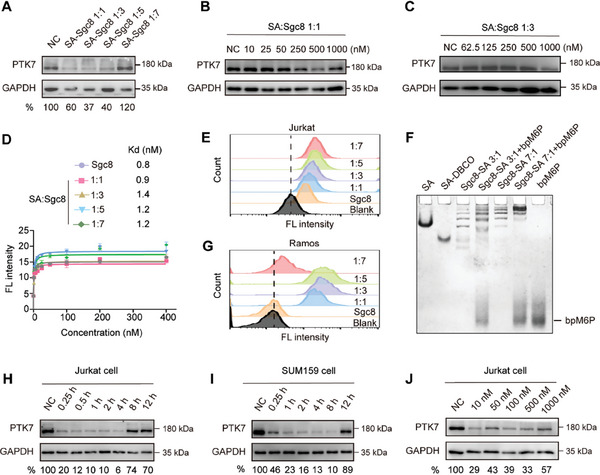
PTK7‐targeted protein degradation by monovalent and multivalent AptLYTACs. A) Western blot result of PTK7 levels in Jurkat cells treated with 500 nm of AptLYTACs with different ratios of SA:Sgc8 (1:1, 1:3, 1:5, and 1:7) for 4 h. B,C) Western blot analysis of PTK7 after treatment with bpM6P‐Sgc8‐SA (SA:Sgc8 = 1:1) or bpM6P‐Sgc8‐SA (SA:Sgc8 = 1:3) in various concentrations for 2 h in Jurkat cells. D) Binding affinities of FAM‐labeled Sgc8 and FAM‐AptLYTACs (SA:Sgc8 = 1:1, 1:3, 1:5, or 1:7) toward Jurkat cells, as measured by flow cytometry. Data were shown as mean ± SD obtained from three independent experiments. E) Fluorescence intensities of Jurkat cells treated with 200 nm FAM‐Sgc8 or different FAM‐labeled AptLYTACs (SA:Sgc8 = 1:1, 1:3, 1:5, and 1:7) for 0.5 h at room temperature, as measured by flow cytometry. F) Native PAGE analysis of the SA‐Sgc8 (1:3, 1:7) assembled with an equal amount of bpM6P. G) Fluorescence intensities of Ramos cells receiving the same treatments as described in (E). H,I) Western blot analysis of the degradation kinetics of PTK7 after treatment with 500 nm bpM6P‐Sgc8‐SA (SA:Sgc8 = 1:3) for different time periods (1–12 h) in Jurkat cells and SUM159 cells. J) Western blot analysis of PTK7 in Jurkat cells after treatment with bpM6P‐Sgc8‐SA (SA:Sgc8 = 1:3) at various aptamer concentrations (10–1000 nm) for 4 h.

We proceeded to compare the protein degradation efficiencies of monovalent and multivalent AptLYTACs. Western blot of PTK7 revealed that multivalent degraders (SA:Sgc8 = 1:3 or 1:5) achieved higher degradation efficiencies than their monovalent counterpart (**Figure** **3**A). Moreover, we found that multivalent AptLYTACs (SA:Sgc8 = 1:3) required a much lower aptamer concentration than the monovalent one to achieve the same degradation efficiency (Figure 3B,C and Figure S10, Supporting Information). However, counterintuitively, increasing the Sgc8 valency to 7 resulted in reduced degradation efficiency (Figure 3A). To explain this phenomenon, we first examined whether the aptamer valency of Sgc8‐SA conjugates could affect their binding affinities to target cells. Cytometric analysis showed that the dissociation constant (Kd) values of various AptLYTACs (SA:Sgc8 = 1:1, 1:3, 1:5, and 1:7) to Jurkat cells were not significantly changed compared to that of free Sgc8 (Figure 3D), which was further supported by their similar cell binding abilities (Figure 3E). We then asked whether the excessive conjugation of Sgc8 blocked the engagement of bpM6P to SA. Gel electrophoresis analysis revealed that high‐valency SA‐Sgc8 (1:7) exhibited a comparable bpM6P‐assembling efficiency as trivalent SA‐Sgc8 (Figure 3F). We further examined the CI‐M6PR‐mediated uptake efficiencies of different AptLYTACs in PTK7‐negative Ramos cells, which precludes the effect of Sgc8 on cellular uptake. Flow cytometric analysis uncovered that AptLYTACs (SA:Sgc8 = 1:7) showed a drastically reduced level of endocytosis compared with other formulations (Figure 3G). Collectively, the above results suggest that at optimized SA:aptamer ratios, multivalent AptLYTACs have the advantage of the so‐called “handle effect” to bind and transport more than one target protein into lysosomes simultaneously,^[^ ^25^ ^]^ but excessive aptamers in AptLYTACs may impair the engagement between bpM6P and CI‐M6PR and reduce protein degradation efficiencies instead.

We next evaluated the degradation kinetics of PTK7 in both Jurkat and SUM159 cells following treatment with bpM6P‐Sgc8‐SA (SA:Sgc8 = 1:3) AptLYTACs over a time course. Our results demonstrated that PTK7 degradation is significantly initiated at 15 min post‐AptLYTACs incubation and reaches its peak at 4 h, as detected by Western blot analysis (Figure 3H,I). However, prolonged incubation time resulted in decreased protein degradation efficiency in a reversible manner. We hypothesized that the fast degradation kinetics of AptLYTACs might be attributed to the intrinsic susceptibility of oligonucleotides toward environmental nucleases, as AptLYTACs were incubated with cells in fetal bovine serum (FBS)‐supplemented cell culture media. As expected, gel electrophoresis results indicate a gradual degradation of bpM6P‐Sgc8‐SA in FBS‐containing solutions within 12 h (Figure S11, Supporting Information). However, outside the scope of this work, the nuclease resistance of oligonucleotides can be readily achieved using well‐established strategies, such as the incorporation of synthetic nucleic acid analogs.^[^ ^26^, ^27^ ^]^ Furthermore, we did not observe a concentration‐dependent “hook effect” in the AptLYTACs system (Figure 3J), which is consistent with previous findings in LYTACs.^[^ ^12^ ^]^ Moreover, we considered the bpM6P to Sgc8‐SA concentration ratio, and our results indicated that a fourfold molar excess of bpM6P to Sgc8‐SA yielded the most efficient outcome (Figure S12, Supporting Information). Together, based on our results and analyses, we conclude that trivalent AptLYTACs are optimal for targeted protein degradation in our system.

### 2.3 SL1‐Conjugated Trivalent AptLYTAC for Met‐Targeted Degradation

**Figure 4:**
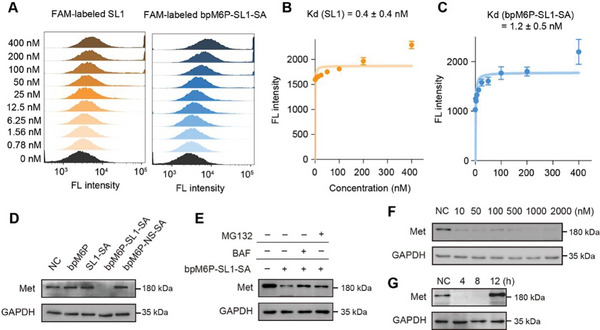
Targeted degradation of Met by multivalent AptLYTACs. A) Flow cytometric results showing the binding abilities of SL1 aptamer and bpM6P‐SL1‐SA in MDA‐MB‐231 cells at 4 °C. B,C) Binding affinity analysis of SL1 aptamer and bpM6P‐SL1‐SA in MDA‐MB‐231 cells. Data were shown as mean ± SD obtained from three independent experiments. D) Western blot result of Met‐targeted degradation by multivalent AptLYTACs (SA:SL1 = 1:3) in MDA‐MB‐231 cells after treatment without (NC) or with SL1‐SA (500 nm), bpM6P‐SL1‐SA (bpM6P 2 µm, SL1‐SA 500 nm), bpM6P‐NS‐SA (bpM6P 2 µm, NS‐SA 500 nm, NS stands for negative sequence), or bpM6P alone (2 µm) for 4 h. E) Western blot result of Met levels in MDA‐MB‐231 cells that were pretreated with 100 nm BAF or 1 µm MG132 for 2 h and then with bpM6P‐SL1‐SA for 2 h under the maintenance of corresponding inhibitor. F,G) Western blot results of Met levels in MDA‐MB‐231 cells treated with multivalent AptLYTACs with different concentrations of bpM6P‐SL1‐SA for 4 h (F) or with bpM6P‐SL1‐SA for different periods of time as indicated (G).

To expand the applicability of our approach to other therapeutically significant membrane‐associated proteins, we extended our investigation on trivalent AptLYTACs targeting the hepatocyte growth factor receptor (Met), a crucial player in tumor growth and metastasis.^[^ ^28^ ^]^ The successful synthesis and stability of SL1 (a Met‐targeting aptamer) and bpM6P‐SL1‐SA were confirmed (Figures S13 and S14, Supporting Information). Meanwhile, their binding affinities to cells were carefully characterized (**Figure** **4**A–C). Like PTK7 degradation, a dramatic reduction in the expression level of Met was observed in MDA‐MB‐231 cancer cells after treatment with AptLYTACs (Figure 4D). Notably, AptLYTACs demonstrated efficient Met degradation across a wide range of concentrations in MDA‐MB‐231cells (10 nm to 2 µm, Figure 4F) and SUM159 cells (10 nm to 1 µm, Figure S15, Supporting Information). Furthermore, we confirmed that the action of degradation complied with the lysosome‐mediated pathway (Figure 4E). Through dynamic profiling of Met protein levels, we observed a substantial decline at 4 and 8 h post‐treatment, but interestingly, the protein level rebounded to baseline at 12 h post‐treatment (Figure 4G). This temporal pattern of degradation is consistent with the mode observed for PTK7 degradation and may share a similar mechanism (Figure 3H).

### 2.4 Bi‐Specific AptLYTACs Simultaneously Degraded PTK7 and Met

**Figure 5:**
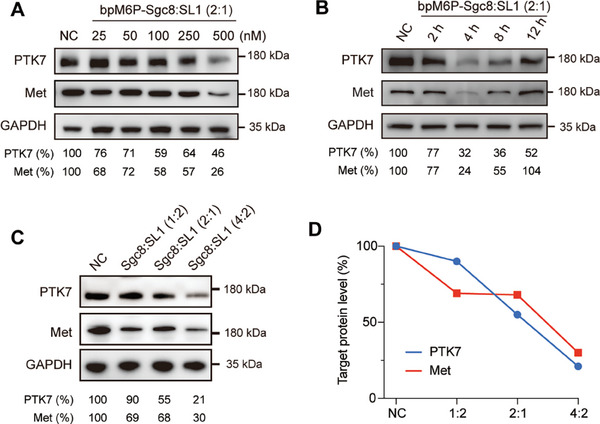
Simultaneous degradation of PTK7 and Met by the bi‐specific multitarget AptLYTACs. A) Western blot result of dual‐targeted degradation by bi‐specific AptLYTACs (Sgc8:SL1 = 2:1, SA:aptamer = 1:3) after treatment without AptLYTACs (NC) or with different concentrations of bpM6P‐Sgc8‐SL1‐SA for 4 h in SUM159 cells. B) PTK7 and Met expression in SUM159 cells after treatment with bpM6P‐Sgc8‐SL1‐SA (Sgc8:SL1 = 2:1) for different periods of time as indicated. C) Western blot result of SUM159 cells after being treated without (NC) or with bi‐specific AptLYTACs with different aptamer loading ratios (Sgc8:SL1:SA = 1:2:1, 2:1:1, and 4:2:1) for 4 h. D) Quantitative result of grayscale analysis of (C).

Considering the complexities of multifactorial diseases, the concept of single‐molecule multitarget degraders, capable of concurrently targeting different proteins, holds promise in overcoming drug–drug interactions, thereby reducing side effects and improving therapeutic efficacy.^[^ ^29^ ^]^ Building upon the successful targeting of protein degradation using multivalent AptLYTACs, we sought to develop bi‐specific degraders capable of simultaneously degrading two different protein targets to elicit synergistic effects akin to dual PROTACs.^[^ ^30^, ^31^ ^]^ To validate this concept, we designed and synthesized multitarget AptLYTACs to concurrently induce the degradation of PTK7 and Met. The successful synthesis of multitarget AptLYTACs was carefully characterized by combining both Sgc8 and SL1 aptamers with SA at different ratios (SA: aptamer = 1:3, Sgc8:SL1 = 1:1, 1:2, and 2:1) (Figure S16, Supporting Information). Our results revealed that the 2:1 ratio of Sgc8:SL1 achieved maximal degradation of both target proteins (Figure S17, Supporting Information). Furthermore, we demonstrated its degradation efficacy over a wide range of concentrations (25 to 500 nm) in SUM159 cells (**Figure** **5**A) and MDA‐MB‐231 cells (Figure S18, Supporting Information). To understand the kinetics of PTK7 and Met degradation, we treated SUM159 cells with 250 nm of multitarget AptLYTACs for various durations (2, 4, 8, and 12 h) (Figure 5B). Furthermore, to enhance dual‐target protein degradation, we constructed various bi‐specific AptLYTACs with varied Sgc8:SL1:SA ratios, ranging from 2:1:1 to 4:2:1 (Figure S19, Supporting Information). As anticipated, higher aptamer loading conferred improved degradation efficiencies for each protein target (Figure 5C,D). Our data collectively demonstrate that the bi‐specific multivalent AptLYTACs offer a promising platform for dual‐target degradation, exhibiting high potency and selectivity.

## 3 Discussion

In summary, we developed a novel multivalent aptamer‐based lysosome‐targeting chimera (AptLYTAC) system for targeted protein degradation. It is noteworthy that the simultaneous binding of multivalent aptamers or polyvalent antibodies to multiple targets has been widely exploited to enhance targeting efficiency.^[^ ^32^, ^33^ ^]^ The benefit of this multi‐target inhibition approach has been validated in the synergetic blocking virus strategy, where multi‐heterogeneous aptamers inhibit the infection of both wild‐type and various SARS‐CoV‐2 mutant strains.^[^ ^34^ ^]^ In this study, we have demonstrated that the multivalent LYTACs exhibit greater efficacy in degrading proteins compared to their monovalent counterparts. This may be attributed to the greater conformational diversity of multivalent AptLYTACs, allowing them to more effectively access proteins that require specific folded binding conformations or to simultaneously bind to multiple target proteins. It is important to note that excessive aptamer loading leading to steric hindrance may affect the binding between different targets; therefore, the aptamer valency in AptLYTACs should be carefully optimized.

Importantly, this study represents the first successful application of the lysosome‐targeting chimera system for the simultaneous degradation of two proteins. Leveraging the ease of modification and multivalent characteristics of aptamers, we may have the potential to achieve simultaneous degradation of multiple important proteins. In conclusion, our findings provide valuable insights into the advantages of multivalent aptamers for targeted protein degradation and offer new avenues for the design of more potent degraders with potential clinical applications.

## 4 Experimental Section

### Chemicals, Cell Lines, and Reagents

DNA sequences, Gel‐red DNA staining dye, SDS, Tris‐borate EDTA buffer, 30% polyacrylamide, TEMED, and APS used in this study were procured from Sangon Biotech (Shanghai, China). Urea, DPBS, bovine serum albumin (BSA), Glucose, and Tween 20 were purchased from Sigma‐Aldrich (USA). HtDNA (D8050) was obtained from Solarbio, while DBCO‐NHS (HY‐42973) was procured from MedChemExpress (MCE). Streptavidin (NRPA18S) was obtained from NUPTEC, and FITC‐SA was obtained from Bioss. LysoTracker Red and Hoechst 33342 were purchased from Beyotime. PTK7 antibody (25618S) and Met antibody (8198S) were obtained from Cell Signaling Technology. HRP‐conjugated Goat Anti‐Rabbit IgG (WLA023) was obtained from WanLeibio, and GAPDH antibody (ET1601‐4) was obtained from HUABIO. All cell lines were obtained from ATCC, and cell cultures were prepared according to ATCC formulations. Specifically, SUM159 cells, Ramos cells, and Jurkat cells were cultured in RPMI‐1640 medium supplemented with 10% FBS and 100 U mL^−1^ penicillin‐streptomycin and maintained at 37 °C in a 5% CO_2_ atmosphere. MDA‐MB‐231 cells were cultured in DMEM medium with 10% FBS and 100 U mL^−1^ penicillin‐streptomycin and maintained at 37 °C under a 5% CO_2_ atmosphere. All media used for cell culture and FBS were purchased from Gibco (Waltham, MA, USA), while penicillin‐streptomycin was obtained from Hyclone (Waltham, MA, USA). The washing buffer used to remove excess aptamers was made by mixing 4.5 g L^−1^ glucose and 5 mm MgCl_2_ in DPBS with calcium chloride and magnesium chloride. A binding buffer, designed to minimize the nonspecific binding of aptamers to cells, was prepared by adding HtDNA (5 mg mL^−1^) and BSA (1 mg mL^−1^) in the washing buffer.

### SA Labeling with DBCO‐NHS

200 µL of a 100 mg mL^−1^ SA solution was diluted with 3 mL of freshly prepared DBCO‐NHS solution (withdrawn from 25.5 µL of 100 mm in DMSO). The resulting mixture was incubated at 4 °C overnight with slow rotation. Finally, the reaction mixture was subjected to ten rounds of filtration and washing using a 500 µL, 30 kDa Amicon Centrifugal Filter to obtain SA‐DBCO. Then, the numbers of DBCO clicked on SA were quantified by Bruker RapifleX MALDI‐TOF/TOF MS.

### Procedure for Sgc8/SL1‐SA Conjugates

To prepare the Sgc8/SL1‐SA conjugates, a 100 µm solution of Sgc8/SL1 aptamer (at 1.5, 4.5, 7.5, or 15 equivalents) was added to SA‐DBCO (100 µg in 50 µL of DPBS) to allow incubation at 4 °C overnight with gentle rotation. The resulting reaction mixture was filtered ten times using a 50 kDa Amicon Centrifugal Filter and resuspended in 100 µL of 1× DPBS to obtain a solution in DPBS. The SA concentration of the Sgc8/SL1‐SA conjugates was determined using a bicinchoninic acid (BCA) assay, while the DNA concentration was analyzed by gel imaging.

### Procedure for the Bi‐Specific Multivalent AptLYTACs

To prepare the bi‐specific multivalent AptLYTACs, 100 µm solutions of Sgc8 and SL1 aptamer (Sgc8:SL1 = 1:1, 1:2, 2:1, and 4:2) were added to SA‐DBCO (SA:aptamer = 1:3) solutions and allowed to incubate at 4 °C overnight with gentle rotation. The resulting reaction mixture was filtered ten times using a 50 kDa Amicon Centrifugal Filter and resuspended in 100 µL of 1× DPBS to obtain a solution in DPBS. The SA concentration of the bi‐specific multivalent AptLYTACs was determined using a BCA assay, while the DNA concentration was analyzed by gel imaging.

### Procedure for bpM6P‐Sgc8/SL1/Bi‐Specific Aptamer‐SA Conjugates

To prepare the bpM6P‐Sgc8/SL1‐SA conjugates, a 0.5 µm solution of Sgc8/SL1‐SA/bi‐specific aptamer (SA:Sgc8/SL1/ bi‐specific aptamer = 1:3) was added to various concentrations (0–2 µm) of bpM6P (100 mm in DPBS) and allowed to incubate at 4 °C for 30 min with gentle rotation. The formation of bpM6P‐Sgc8/SL1/bi‐specific aptamer‐SA conjugates was analyzed by 10% native polyacrylamide gel electrophoresis (PAGE) or 4–20% SDS‐PAGE.

### Flow Cytometric Analysis of Streptavidin Uptake

Jurkat cells (≈5 × 10^5^) were collected and centrifugated for 3 min at 1200 rpm, 4 °C, to remove the culture media. Then, the harvested cells were washed twice with DPBS. Jurkat cells were incubated with or without bpM6P and FITC‐SA for 1 h at room temperature. Subsequently, cells were washed twice to eliminate unbound samples and resuspended in 200 µL of 1× DPBS. The final cell samples were submitted to flow cytometry (BD FACSVerse, USA). The obtained fluorescence distribution statistics were analyzed by FlowJo software.

### Confocal Microscopy Analysis of Streptavidin Uptake

Jurkat cells (≈5 × 10^5^) were harvested by centrifugation at 1200 rpm, 4 °C, for 3 min to remove culture media. Afterward, cells were washed twice with DPBS and plated in a confocal dish. The cells were then treated with bpM6P and FITC‐SA, or left untreated as a control, for 4 h at room temperature. The cells were then washed twice to remove any unbound samples and resuspended in 200 µL of DPBS, followed by co‐staining with LysoTracker Red and Hoechst 33342 (2.5 µg mL^−1^) for 10 min at room temperature, respectively. Subsequently, cells were washed three times to eliminate unbound samples and were imaged by a confocal laser scanning microscope (NIKON, A1 HD25) at ×63 magnification under oil.

### Flow Cytometric Analysis of Specific Binding of Sgc8 and bpM6P‐Sgc8‐SA

Jurkat and Ramos cells (≈5 × 10^5^) were subjected to two rounds of washing with 200 µL of washing buffer to remove the culture media. Then, the cells were incubated with 100 nm FAM‐NS, FAM‐Sgc8, FAM‐Sgc8‐SA (SA:Sgc8 = 1:3), and FAM‐bpM6P‐Sgc8‐SA (SA:Sgc8 = 1:3) in 200 µL of binding buffer, while maintained on ice for 30 min, respectively. The cells were later washed twice with washing buffer and resuspended in 200 µL of binding buffer. The final cell samples were submitted to flow cytometry (BD FACSVerse, USA). The obtained fluorescence distribution statistics were analyzed by FlowJo software.

### PTK7 Uptake Experiment Using Confocal Microscopy

Jurkat cells (≈5 × 10^5^) were seeded in a confocal dish after washing twice with washing buffer. The cells were then incubated with FAM‐Sgc8 or FAM‐bpM6P‐Sgc8‐SA (at a concentration of 0.5 nm) for 4 h at room temperature. After incubation, cells were washed three times with washing buffer and then co‐stained with LysoTracker Red and Hoechst 33342 (at a concentration of 2.5 µg mL^−1^) for 10 min at room temperature. The live cells were imaged using a confocal laser scanning microscope (NIKON, A1 HD25) at ×63 magnification under oil.

### Target Protein Degradation Experiment Using Western Blot

To evaluate membrane PTK7 degradation, Jurkat cells (≈1 × 10^6^) were seeded into a 6‐well dish 1 day prior to the experiment. The cells were then treated with Sgc8‐SA (500 nm), bpM6P‐Sgc8‐SA (bpM6P 2 µm, Sgc8‐SA 500 nm), bpM6P‐NS‐SA (bpM6P 2 µm, NS‐SA 500 nm), and bpM6P alone (2 µm), or left untreated as negative control (NC), for 4 h. To investigate the degradation kinetics of PTK7, Jurkat cells or SUM159 cells (≈1 × 10^6^) were treated with 500 nm of bpM6P‐Sgc8‐SA (SA:Sgc8 = 1:3) for different time periods ranging from 1 to 24 h. To determine the concentration‐dependent degradation of PTK7, Jurkat cells (≈1 × 10^6^) were treated with bpM6P‐Sgc8‐SA (SA:Sgc8 = 1:1 or 1:3) at concentrations ranging from 10 to 1000 nm for 4 h. To assess the effect of bpM6P concentration on PTK7 degradation, Jurkat cells (≈1 × 10^6^) were treated with Sgc8‐SA (0.5 µm), along with varying concentrations of bpM6P (0–2 µm), for 4 h. To evaluate the role of lysosomal pathways in PTK7 degradation, Jurkat cells (≈1 × 10^6^) were pretreated with 100 nm bafilomycin A1 or 1 µm MG132 for 2 h and then treated with 500 nm bpM6P‐Sgc8‐SA (SA:Sgc8 = 1:3) for another 2 h with the maintenance of the corresponding inhibitor. To compare PTK7 degradation efficiencies of monovalent and multivalent AptLYTACs, Jurkat cells (≈1 × 10^6^) were treated with 500 nm of bpM6P‐Sgc8‐SA (SA:Sgc8 = 1:1, 1:3, 1:5 and 1:10) for 4 h. To evaluate the degradation of Met, MDA‐MB‐231 cells (≈1 × 10^6^) were seeded in a 6‐well dish 1 day prior to the experiment. Cells were then treated with SL1‐SA (500 nm), bpM6P‐SL1‐SA (bpM6P 2 µm, SL1‐SA 500 nm), bpM6P‐NS‐SA (bpM6P 2 µm, NS‐SA 500 nm), and bpM6P alone (2 µm), or left untreated as negative control, for 4 h.

The degradation kinetics of Met was analyzed by treating MDA‐MB‐231 cells (≈1 × 10^6^) with 500 nm of bpM6P‐SL1‐SA (SA:SL1 = 1:3) for varying time periods ranging from 1 to 24 h. The concentration‐dependent degradation of Met was determined by treating MDA‐MB‐231 cells (≈1 × 10^6^) with bpM6P‐SL1‐SA (SA:SL1 = 1:3) at concentrations ranging from 10 to 2000 nm for 4 h, and was also measured in SUM159 cells (≈1 × 10^6^) treating with bpM6P‐SL1‐SA (SA:SL1 = 1:3) at a wide range of concentrations (10 to 1000 nm) for 4 h. To assess the role of lysosomal pathways in Met degradation, MDA‐MB‐231 cells (≈1 × 10^6^) were pretreated with 100 nm bafilomycin A1 or 1 µm MG132 for 2 h and then treated with 500 nm bpM6P‐SL1‐SA (SA:SL1 = 1:3) for another 2 h with the maintenance of the corresponding inhibitor.

To compare PTK7 and Met degradation efficiencies of bi‐specific multivalent AptLYTACs, SUM159 cells (≈1 × 10^6^) were treated with 250 nm of bpM6P‐bi‐specific aptamers‐SA (Sgc8:SL1 = 1:1, 1:2, and 2:1, SA:aptamer = 1:3) for 4 h. To compare PTK7 and Met degradation efficiencies of bi‐specific multivalent AptLYTACs with different aptamer loading contents, SUM159 cells (≈1 × 10^6^) were treated with 250 nm of bpM6P‐bi‐specific aptamers‐SA (Sgc8:SL1:SA = 1:2:1, 2:1:1 and 4:2:1) for 4 h. To investigate the degradation kinetics of PTK7 and Met, SUM159 cells (≈1 × 10^6^) were treated with 500 nm of bpM6P‐bi‐specific aptamers‐SA (Sgc8:SL1 = 2:1, SA:aptamer = 1:3) for different time periods ranging from 2 to 12 h. To determine the concentration‐dependent degradation of PTK7 and Met, SUM159 cells and MDA‐MB‐231 cells (≈1 × 10^6^) were treated with bpM6P‐bi‐specific aptamers‐SA (Sgc8:SL1 = 2:1, SA:aptamer = 1:3) at concentrations ranging from 25 to 500 nm or 50 to 500 nm for 4 h.

After different sample treatments as indicated, cells were harvested and concentrated using a washing buffer before protein concentration was determined using BCA assays. Equal amounts of lysate (20 µg) were loaded onto a 10% SDS‐PAGE and transferred to a polyvinylidene fluoride (PVDF) membrane. To reduce nonspecific binding, the membrane was blocked with 5% skim milk powder dissolved in 1×TBST (0.05% Tween‐20) for 1 h. Then, the membrane was incubated with a primary antibody (diluted 1:1000 with TBST) overnight at 4 °C, followed by 3× washing with TBST. Subsequently, the membrane was incubated with a secondary antibody (diluted 1:5000 with TBST) for 1 h at room temperature and washed three times with TBST. The membrane was incubated in ECL reagent (Biosharp, Beijing) substrate for 3–5 min before imaging (ImageQuant 800, AMERSHAM). Grayscale values of images were analyzed by Image J.

### Flow Cytometric Analysis of Sgc8 and AptLYTACs Uptake

Jurkat and Ramos cells (≈5 × 10^5^) were harvested and centrifuged at 1200 rpm, 4 °C, for 3 min to remove the culture media. Cells were washed twice with washing buffer before being incubated with 200 nm of FAM‐Sgc8 and FAM‐bpM6P‐Sgc8‐SA (SA:Sgc8 = 1:1, 1:3, 1:5 or 1:7) for 0.5 h at room temperature. After incubation, cells were washed twice to remove unbound samples and resuspended in 200 µL of washing buffer. The fluorescence of the final cell samples was detected by a BD FACSVerse flow cytometer. The obtained fluorescence distribution statistics were analyzed by FlowJo software.

### Binding Affinity Analysis of AptLYTACs

Jurkat cells or MDA‐MB‐231 cells (≈5 × 10^5^) were subjected to two rounds of washing with 200 µL of washing buffer. Following that, the cells were separately exposed to different concentrations of FAM‐labeled Sgc8/SL1, FAM‐bpM6P‐Sgc8‐SA (SA:Sgc8 = 1:1, 1:3, 1:5, or 1:7), or FAM‐labeled bpM6P‐SL1‐SA (SA:Sgc8 = 1:3) in 200 µL of binding buffer while kept on ice for 30 min. Cells were later washed twice with washing buffer, centrifuged at 1200 rpm for 3 min, and resuspended in 200 µL of binding buffer. Binding affinity was assessed using the geometric mean of fluorescence intensity of Sgc8 and AptLYTACs. Kd values were computed by fitting the dependence of fluorescence intensity (Y) and the concentrations of Sgc8 and AptLYTACs (X) into the one‐site saturation equation Y = Bmax X/ (Kd + X), utilizing GraphPad Prism 8.0.

### Serum Stability Test of AptLYTACs

Sgc8, SL1, bpM6P‐Sgc8 ‐SA (SA:Sgc8 = 1:1, 1:3 or 1:7), or bpM6P‐SL1 ‐SA (SA:SL1 = 1:3) at a concentration of 1 µm were incubated with 20% FBS of RPMI‐1640 medium for different periods of time (1–24 h) at 37 °C. After incubation, the samples were loaded onto an 8% denaturing polyacrylamide gel in 1×TBE‐Mg buffer and run at 400 V for 15 min. The gel was then stained with Gel Red staining solution before imaging. The grayscale values of the images were analyzed by ImageJ.

### Statistical Analysis

Statistical analysis was performed using GraphPad Prism version 8.0. One‐site saturation equation Y = Bmax X/ (Kd + X) was used to calculate Kd. Data were expressed as mean ± SD and sample size (*n*) for each statistical analysis is represented in the corresponding figure legends.

## Conflict of Interest

The authors declare no conflict of interest.

## Author Contributions

Q.D. and H.‐R.J. contributed equally to this work. W.T., Q.W., H.‐R.J., and Y.W. conceived, supervised, and acquired funding for the study. Q.D., H.‐R.J., and Q.W. designed the experiments. Q.D. and H.‐R.J. performed most of the experiments and data analysis with the help of W.C., C.Q., K.Z., F.J., T.F., Y.W., and M.F. The manuscript was written by H.‐R.J. and Q.D., and revised by Q.W. and W.T. All the authors contributed to the discussion of the research and manuscript preparation.

## Supporting information

Supporting Information

## Data Availability

The data that support the findings of this study are available from the corresponding author upon reasonable request.

## References

[1] C. Peng , J. Q. Trojanowski , V. M. Lee , Nat. Rev. Neurol. 2020, 16, 199. 32203399 10.1038/s41582-020-0333-7 PMC9242841

[2] J. P. Overington , B. Al‐Lazikani , A. L. Hopkins , Nat. Rev. Drug Discovery. 2006, 5, 993. 17139284 10.1038/nrd2199

[3] L. Zhong , Y. Li , L. Xiong , W. Wang , M. Wu , T. Yuan , W. Yang , C. Tian , Z. Miao , T. Wang , S. Yang , Signal Transduction Targeted Ther. 2021, 6, 201. 10.1038/s41392-021-00572-w PMC8165101 34054126

[4] P. J. Carter , G. A. Lazar , Nat. Rev. Drug Discovery. 2018, 17, 197. 29192287 10.1038/nrd.2017.227

[5] R. Roskoski Jr. , Pharmacol. Res. 2020, 152, 104609. 31862477 10.1016/j.phrs.2019.104609

[6] L. Zhao , J. Zhao , K. Zhong , A. Tong , D. Jia , Signal Transduction Targeted Ther. 2022, 7, 113. 10.1038/s41392-022-00966-4 PMC8977435 35379777

[7] M. Bekes , D. R. Langley , C. M. Crews , Nat. Rev. Drug Discovery. 2022, 21, 181. 35042991 10.1038/s41573-021-00371-6 PMC8765495

[8] K. M. Sakamoto , K. B. Kim , A. Kumagai , F. Mercurio , C. M. Crews , R. J. Deshaies , Proc. Natl. Acad. Sci. U. S. A. 2001, 98, 8554. 11438690 10.1073/pnas.141230798 PMC37474

[9] D. Chirnomas , K. R. Hornberger , C. M. Crews , Nat. Rev. Clin. Oncol. 2023, 20, 265. 36781982 10.1038/s41571-023-00736-3 PMC11698446

[10] H. Zhang , Y. Han , Y. Yang , F. Lin , K. Li , L. Kong , H. Liu , Y. Dang , J. Lin , P. R. Chen , J. Am. Chem. Soc. 2021, 143, 16377. 34596400 10.1021/jacs.1c08521

[11] G. Ahn , S. M. Banik , C. R. Bertozzi , Cell Chem. Biol. 2021, 28, 1072. 33770486 10.1016/j.chembiol.2021.02.024 PMC8286304

[12] S. M. Banik , K. Pedram , S. Wisnovsky , G. Ahn , N. M. Riley , C. R. Bertozzi , Nature. 2020, 584, 291. 32728216 10.1038/s41586-020-2545-9 PMC7727926

[13] G. Ahn , S. M. Banik , C. L. Miller , N. M. Riley , J. R. Cochran , C. R. Bertozzi , Nat. Chem. Biol. 2021, 17, 937. 33767387 10.1038/s41589-021-00770-1 PMC8387313

[14] K. Pance , J. A. Gramespacher , J. R. Byrnes , F. Salangsang , J. C. Serrano , A. D. Cotton , V. Steri , J. A. Wells , Nat. Biotechnol. 2023, 41, 273. 36138170 10.1038/s41587-022-01456-2 PMC9931583

[15] A. J. Chirino , A. Mire‐Sluis , Nat. Biotechnol. 2004, 22, 1383. 15529163 10.1038/nbt1030

[16] Y. Miao , Q. Gao , M. Mao , C. Zhang , L. Yang , Y. Yang , D. Han , Angew. Chem., Int. Ed. 2021, 60, 11267. 10.1002/anie.202102170 33634555

[17] Y. Wu , B. Lin , Y. Lu , L. Li , K. Deng , S. Zhang , H. Zhang , C. Yang , Z. Zhu , Angew. Chem., Int. Ed. 2023, 62, e202218106. 10.1002/anie.202218106 36722696

[18] K. Hamada , T. Hashimoto , R. Iwashita , Y. Yamada , Y. Kikkawa , M. Nomizu , Cell Rep. Phys. Sci. 2023, 4, 101296.

[19] K. Chen , J. Zhou , Z. Shao , J. Liu , J. Song , R. Wang , J. Li , W. Tan , J. Am. Chem. Soc. 2020, 142, 12079. 32516525 10.1021/jacs.9b13370

[20] F. Odeh , H. Nsairat , W. Alshaer , M. A. Ismail , E. Esawi , B. Qaqish , A. A. Bawab , S. I. Ismail , Molecules. 2019, 25, 3. 31861277 10.3390/molecules25010003 PMC6982925

[21] Z. Wang , X. Yang , N. Z. Lee , X. Cao , Micromachines. 2022, 13, 436. 35334728 10.3390/mi13030436 PMC8956053

[22] D. Shangguan , Y. Li , Z. Tang , Z. C. Cao , H. W. Chen , P. Mallikaratchy , K. Sefah , C. J. Yang , W. Tan , Proc. Natl. Acad. Sci. U. S. A. 2006, 103, 11838. 16873550 10.1073/pnas.0602615103 PMC1567664

[23] C. Mauvezin , T. P. Neufeld , Autophagy. 2015, 11, 1437. 26156798 10.1080/15548627.2015.1066957 PMC4590655

[24] N. Guo , Z. Peng , Asia Pac. J. Clin. Oncol. 2013, 9, 6. 22897979 10.1111/j.1743-7563.2012.01535.x

[25] M. Gonzalez‐Cuesta , C. Ortiz Mellet , J. M. Garcia Fernandez , Chem. 2020, 56, 5207. 10.1039/d0cc01135e 32322844

[26] S. Ochoa , V. T. Milam , Molecules. 2020, 25, 4659. 33066073 10.3390/molecules25204659 PMC7587394

[27] J. A. Kulkarni , D. Witzigmann , S. B. Thomson , S. Chen , B. R. Leavitt , P. R. Cullis , R. van der Meel , Nat. Nanotechnol. 2021, 16, 630. 34059811 10.1038/s41565-021-00898-0

[28] C. Birchmeier , W. Birchmeier , E. Gherardi , G. F. Vande Woude , Nat. Rev. Mol. Cell Biol. 2003, 4, 915. 14685170 10.1038/nrm1261

[29] A. Salerno , F. Seghetti , J. Caciolla , E. Uliassi , E. Testi , M. Guardigni , M. Roberti , A. Milelli , M. L. Bolognesi , J. Med. Chem. 2022, 65, 9507. 35816671 10.1021/acs.jmedchem.2c00302 PMC9340767

[30] M. Zheng , J. Huo , X. Gu , Y. Wang , C. Wu , Q. Zhang , W. Wang , Y. Liu , Y. Liu , X. Zhou , L. Chen , Y. Zhou , H. Li , J. Med. Chem. 2021, 64, 7839. 34038131 10.1021/acs.jmedchem.1c00649

[31] J. Dong , J. Miao , Y. Miao , Z. Qu , S. Zhang , P. Zhu , F. Wiede , B. A. Jassim , Y. Bai , Q. Nguyen , J. Lin , L. Chen , T. Tiganis , W. A. Tao , Z. Y. Zhang , Angew. Chem., Int. Ed. 2023, 62, e202303818. 10.1002/anie.202303818 PMC10196813 36973833

[32] L. Wu , H. Ding , X. Qu , X. Shi , J. Yang , M. Huang , J. Zhang , H. Zhang , J. Song , L. Zhu , Y. Song , Y. Ma , C. Yang , J. Am. Chem. Soc. 2020, 142, 4800. 32049531 10.1021/jacs.9b13782

[33] T. Ye , X. Liu , X. Zhong , R. Yan , P. Shi , Nat. Commun. 2023, 14, 5806. 37726299 10.1038/s41467-023-41609-8 PMC10509227

[34] M. Sun , S. Liu , T. Song , F. Chen , J. Zhang , J. A. Huang , S. Wan , Y. Lu , H. Chen , W. Tan , Y. Song , C. Yang , J. Am. Chem. Soc. 2021, 143, 21541. 34855379 10.1021/jacs.1c08226

