# Supplementary material for: Multivalent Aptamer‐Based Lysosome‐Targeting Chimeras (LYTACs) Platform for Mono‐ or Dual‐Targeted Proteins Degradation on Cell Surface
Source: Adv Sci (Weinh). 2024 Feb 29;11(17):2308924. doi: 10.1002/advs.202308924 (PMC11077639; doi:10.1002/advs.202308924)
Supplement: Supplementary file 1 — Supporting Information [file ADVS-11-2308924-s001.pdf]

ADVANCED SCIENCE
Open Access

Supporting Information

for *Adv. Sci.*, DOI 10.1002/advs.202308924

Multivalent Aptamer-Based Lysosome-Targeting Chimeras (LYTACs) Platform for Mono- or Dual-Targeted Proteins Degradation on Cell Surface

Qiao Duan, Hao-Ran Jia, Weichang Chen, Chunhong Qin, Kejing Zhang, Fei Jia, Ting Fu, Yong Wei, Mengyang Fan, Qin Wu and Weihong Tan**

Supporting Information

Multivalent Aptamer-based Lysosome-targeting Chimeras (LYTACs) Platform for Mono- or Dual-targeted Proteins Degradation on Cell Surface

Qiao Duan, Hao-Ran Jia, Weichang Chen, Chunhong Qin, Kejing Zhang, Fei Jia, Ting Fu, Yong Wei, Mengyang Fan, Qin Wu,* and Weihong Tan*

Synthesis and Characterization of Compounds 2-4 and bpM6P.

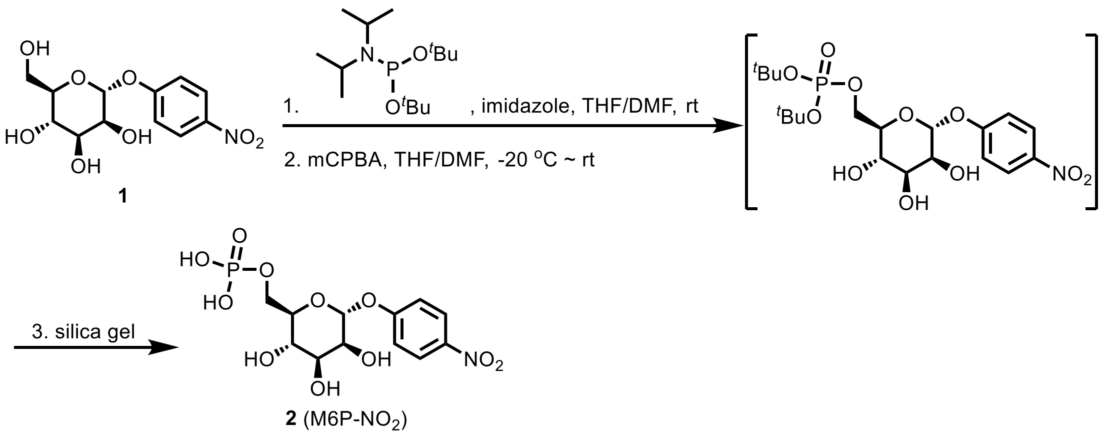

2 (M6P-NO_2)

Compound 2. Commercially available compound **1** (4-nitrophenyl α-D-mannopyranoside) (602 mg, 2.0 mmol, 1.0 eq) was dissolved in dry THF (4 mL) and dry DMF (4 mL). Imidazole (280 mg, 4.0 mmol, 2.0 eq) and di-tert-butyl N, N-diisopropylphosphoramidite (610 mg, 2.2 mmol, 1.1 eq) were added in sequence under room temperature. The mixture was stirred overnight. Then it was cooled to -20 degrees before 70% mCPBA (3-chloroperoxybenzoic acid) (518 mg, 2.1 mmol, 1.05 eq) was added. The mixture was removed from cold bath and returned to room temperature. LC-MS indicated formation of di-tert-butylphosphate of **1**. The di-tert-butylphosphate was easily hydrolyzed through adding some silica gel and stirring awhile (adding some acetic acid if not hydrolyzed completely). The mixture was filtered to remove silica gel, and the filtrate was concentrated before purification via RP-HPLC. Compound **2** (342 mg, 0.90 mmol, 45% yield) was obtained as a colorless oil. ^{1}H NMR (400 MHz, Deuterium Oxide) δ 8.29 (d, J = 9.4 Hz, 2H), 7.32 (d, J = 9.3 Hz, 2H), 5.79 (d, J = 1.9 Hz, 1H), 4.23 (dd, J = 3.5, 1.9 Hz, 1H), 4.18 – 4.04 (m, 3H), 3.92 (t, J = 9.8 Hz, 1H), 3.84 – 3.78 (m, 1H). ^{31}P NMR (162 MHz, Deuterium Oxide) δ 0.46. LCMS [M-H^{+}] = 380.

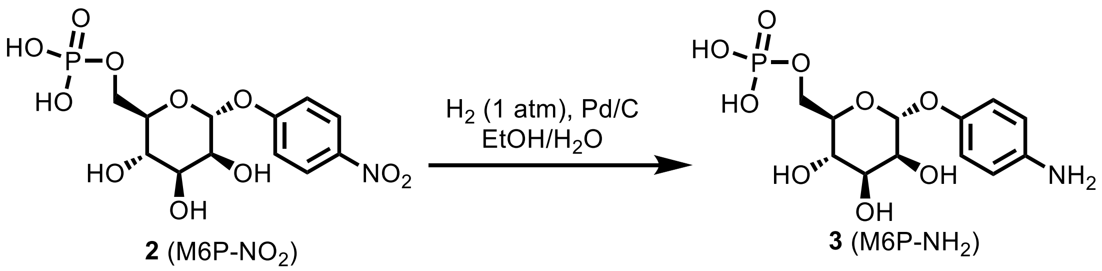

Compound 3. Compound **2** (342 mg, 0.90 mmol) was dissolved in EtOH (4 mL) and water (4 mL) before Pd/C (50 mg) was added. Then a hydrogen balloon was equipped. After evacuation three times, the mixture was stirred and hydrogenated at room temperature overnight. LC-MS indicated the formation of compound **3**, and compound **2** was completely consumed. The mixture was filtered, and the filtrate was concentrated to afford a white solid as compound **3** (203 mg, 0.57 mmol, 64% yield), which was directly used without further purification. ^{1}H NMR (400 MHz, Deuterium Oxide) δ 7.28 (d, *J* = 9.1 Hz, 2H), 7.22 (d, *J* = 9.1 Hz, 2H), 5.57 (d, *J* = 1.8 Hz, 1H), 4.16 (dd, *J* = 3.5, 1.9 Hz, 1H), 4.07 – 3.96 (m, 3H), 3.88 – 3.76 (m, 2H). ^{31}P NMR (162 MHz, Deuterium Oxide) δ 0.56. LCMS [M-H^{+}] = 350.

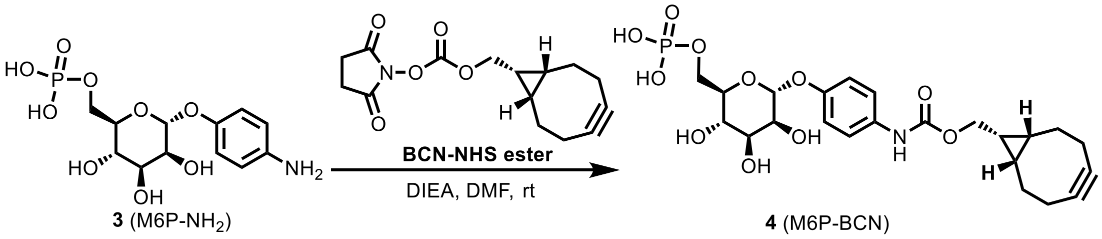

Compound 4. Compound **3** (203 mg, 0.57 mmol, 1.0 eq) and DIEA (221 mg, 1.7 mmol, 3.0 eq) were dissolved in dry DMF (2.0 mL), and then **BCN-NHS ester** (166 mg, 0.57 mmol, 1.0 eq) was added under room temperature. The mixture was stirred overnight. LC-MS indicated the formation of compound **4**, and compound **3** was completely consumed. The mixture was purified via RP-HPLC. Compound **4** (53 mg, 0.10 mmol, 18% yield) was obtained as a pale brown solid. ^{1}H NMR (400 MHz, Deuterium Oxide) δ 7.24 (d, *J* = 8.8 Hz, 2H), 7.07 (d, *J* = 9.2 Hz, 2H), 5.46 (d, *J* = 1.2 Hz, 1H), 4.21 (d, *J* = 8.4 Hz, 2H), 4.09 – 4.08 (m, 1H), 3.98 – 3.90 (m, 3H), 3.84 – 3.78 (m, 2H), 2.26 – 2.13 (m, 6H), 1.55 – 1.52 (m, 2H), 1.40 – 1.36 (m, 1H), 0.93 (t, *J* = 9.2 Hz, 2H). ^{31}P

NMR (162 MHz, Deuterium Oxide) δ 0.46. LCMS [M-H$^+$] = 526.

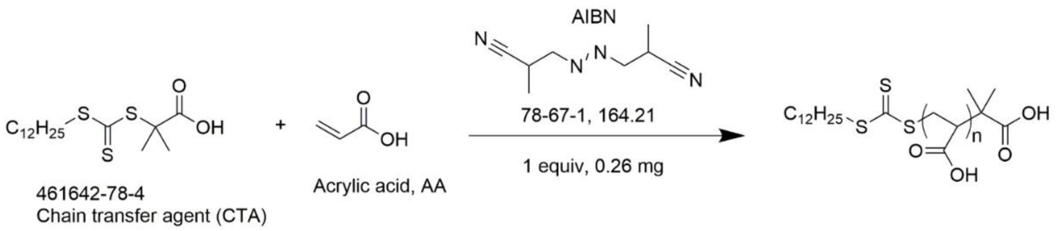

Polymer bp-N3. AA (60 mmol), CTA (1 mmol), and AIBN (0.1 mmol) were added along with 1,4-dioxane (4.5 mL) to a glass vial. The vial was sparged with nitrogen for 30 min and then placed in a preheated oil bath at 70 °C. The reaction was terminated after 5 h by cooling the reaction vial in an ice bath followed by exposure to air. The polymer was then dialyzed against pure water to remove unreacted AA and then lyophilized. MW of the polymer was determined by a DMF GPC.

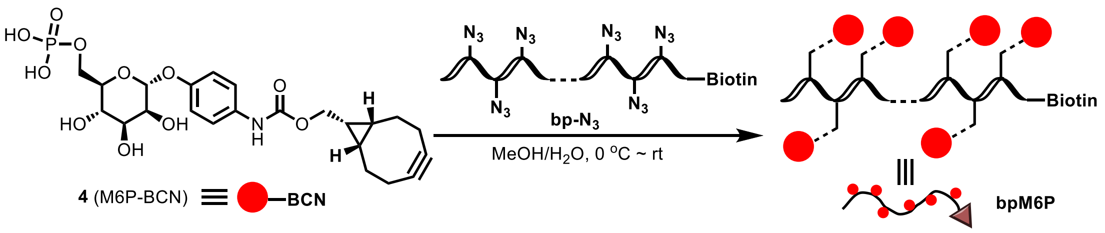

bpM6P. The polymer **bp-N3** (5 mg) was dissolved in MeOH (1 mL). Compound **4** (18 mg) was dissolved in water (4 mL). Then the two solutions were mixed and stirred at room temperature for 1 h. The mixture was dialyzed in water to remove excess compound **4**. The remaining solution in the dialysis bag was lyophilized to afford **bpM6P** (16 mg) as a pale brown powder.

Supporting Figures

A

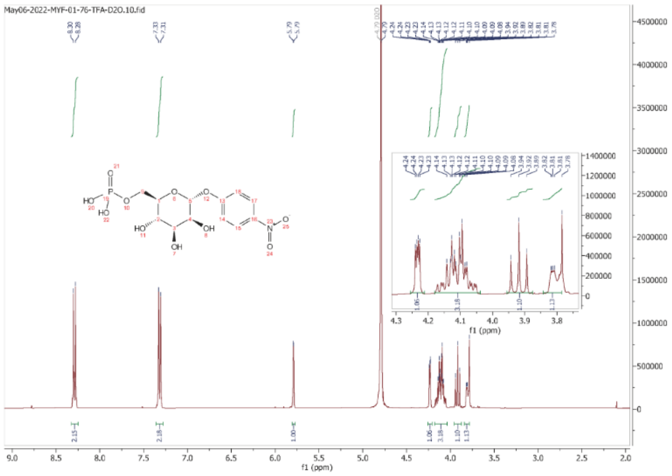

B

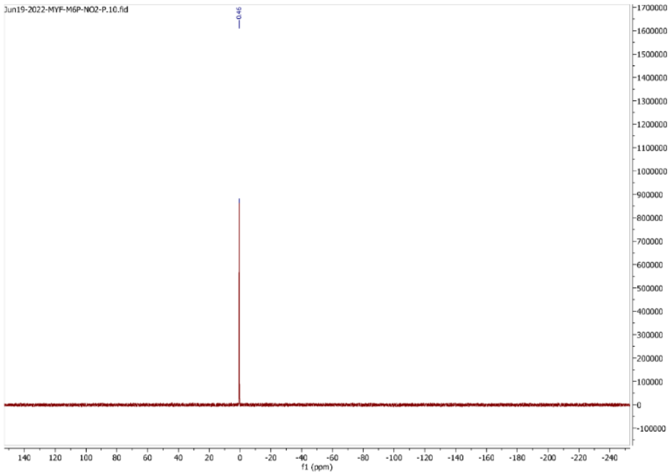

Figure S1. A) ^{1}H NMR and (B) ^{31}P NMR spectra of compound **2**.

A

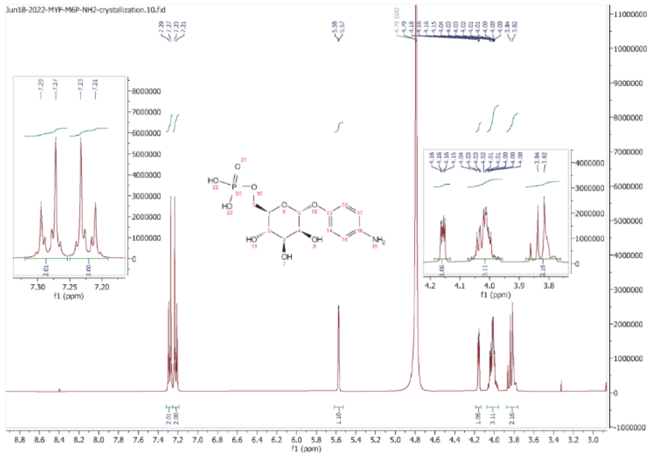

B

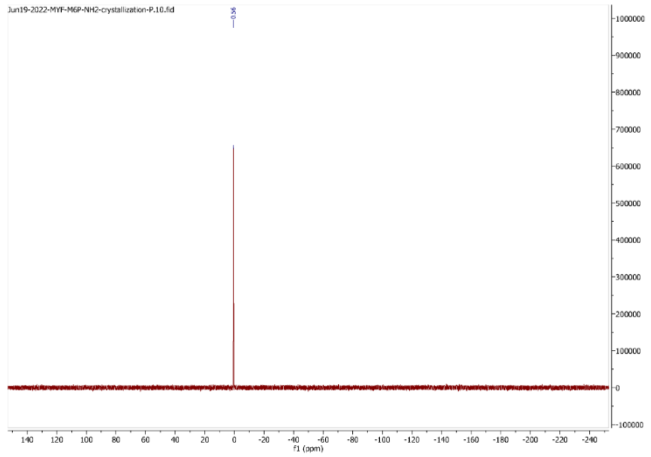

Figure S2. A) ^{1}H NMR and (B) ^{31}P NMR spectra of compound **3**.

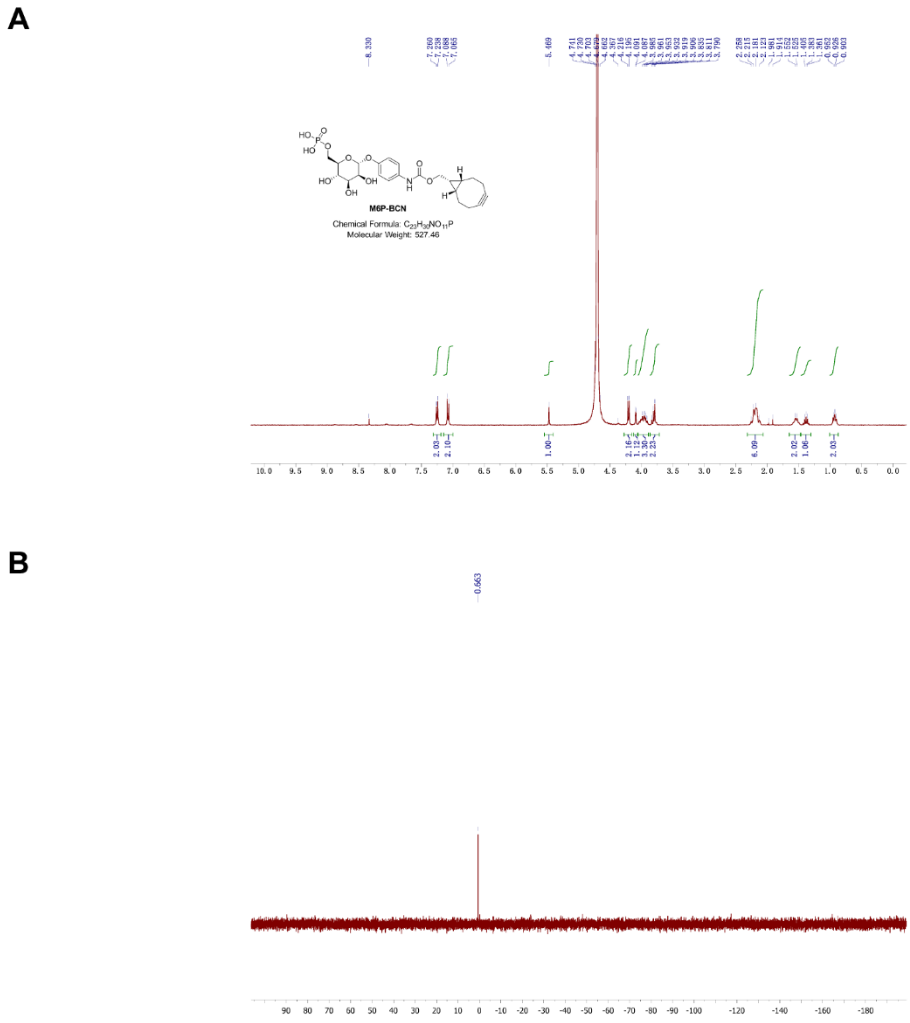

Figure S3. A) ^{1}H NMR and (B) ^{31}P NMR spectra of compound **4**.

A

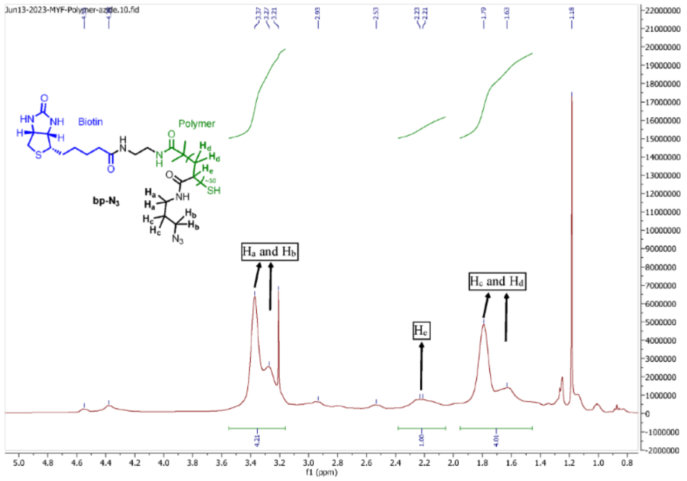

B

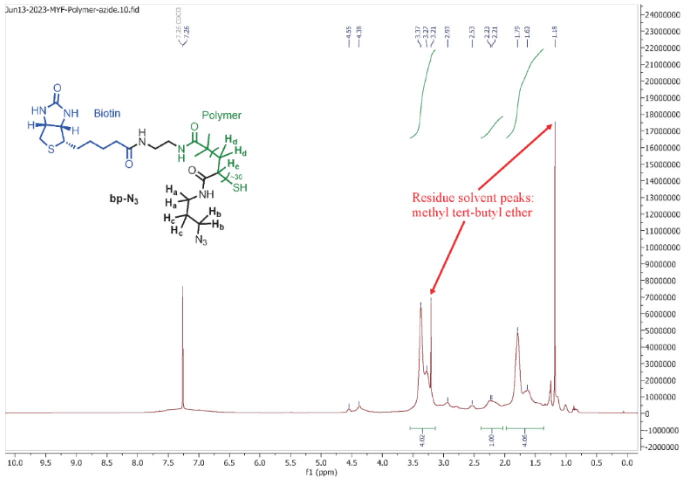

Figure S4. A, B) ^{1}H NMR spectra of bp-N_3.

A

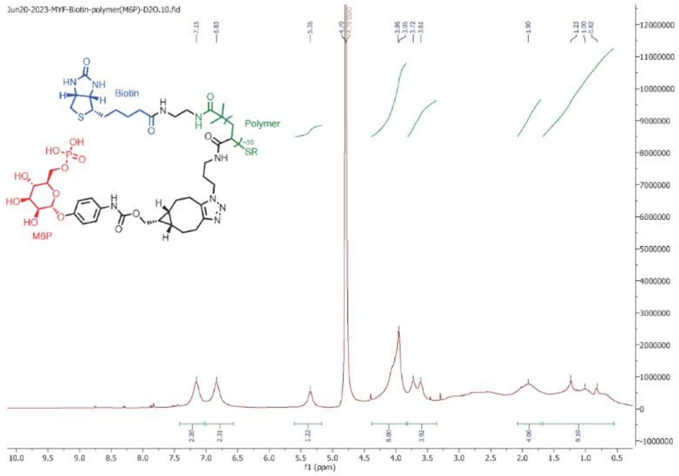

B

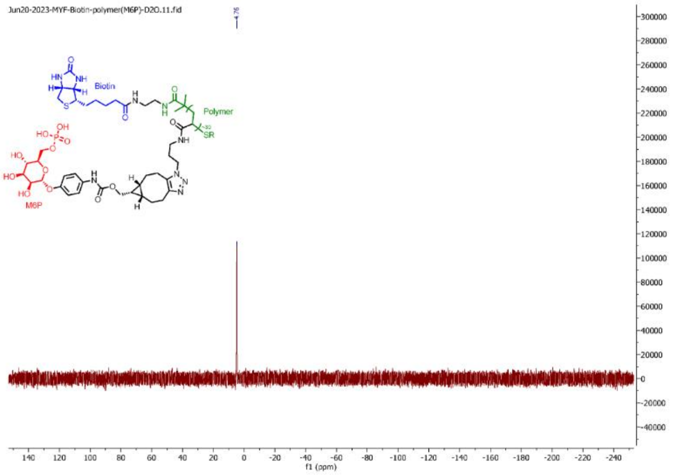

Figure S5. A) ^{1}H NMR and (B) ^{31}P NMR spectra of bpM6P.

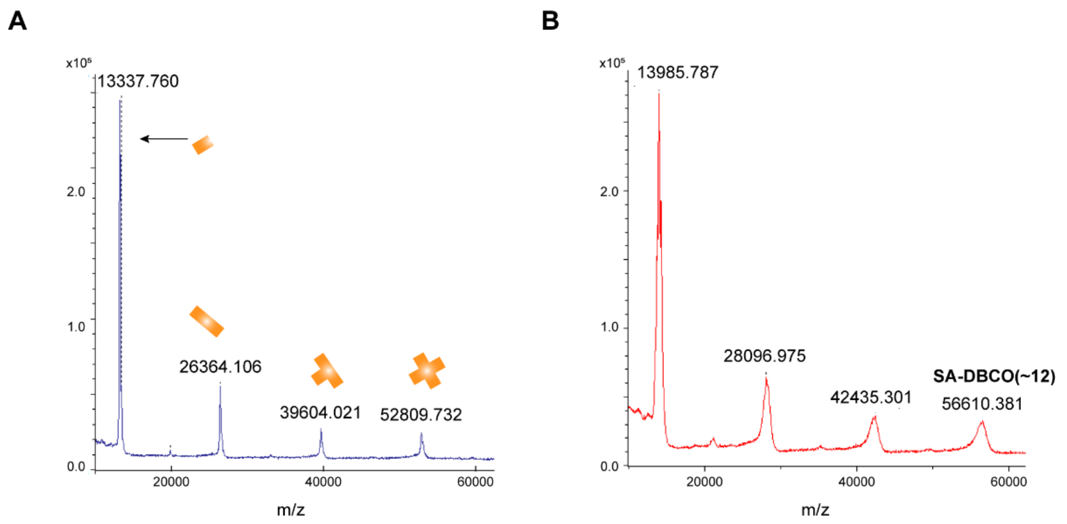

Figure S6. MALDI-TOF mass spectra of SA (A) and SA-DBCO conjugate (B).

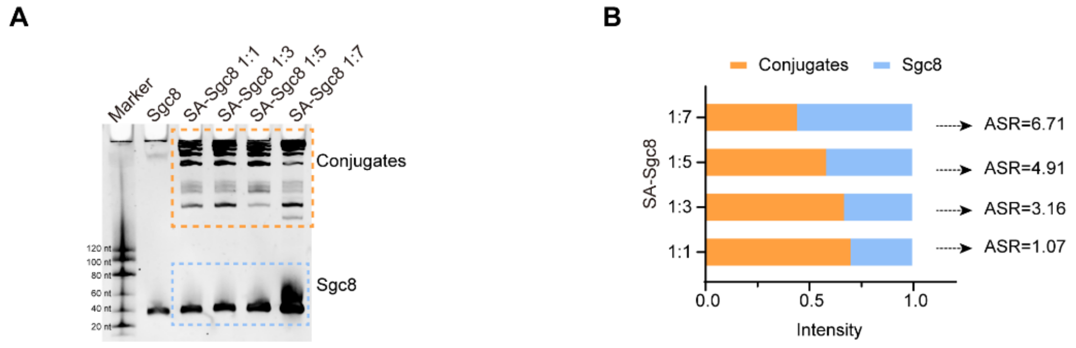

Figure S7. A) Denaturing (7 M urea) gel electrophoresis analysis of the bpM6P-Sgc8-SA conjugates with varied molar ratios under the reaction of SA:Sgc8 (1:1, 1:3, 1:5, and 1:7). B) ImageJ analysis of the left gel. ASR (Aptamer/SA Ratio).

.

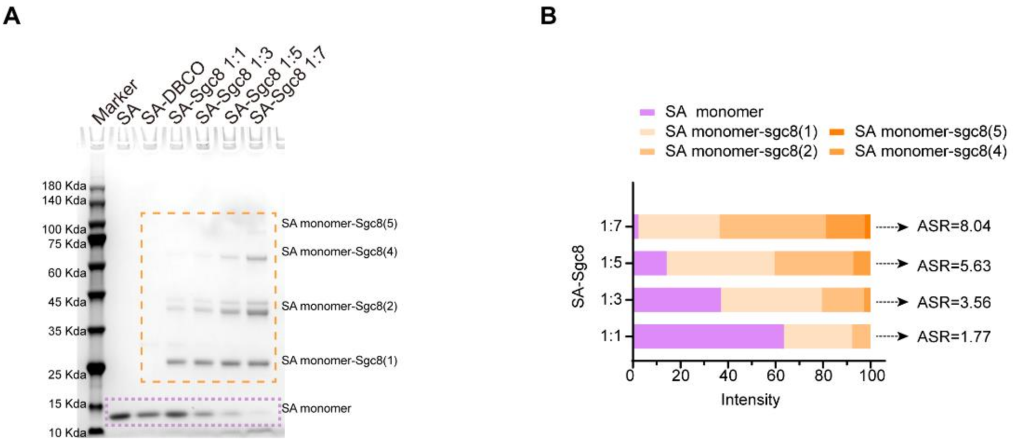

Figure S8. A) 4-20% SDS-PAGE analysis of the bpM6P-Sgc8-SA conjugates with varied molar ratios under the reaction of SA:Sgc8 (1:1, 1:3, 1:5, and 1:7). B) ImageJ analysis of the left gel. ASR (Aptamer/SA Ratio).

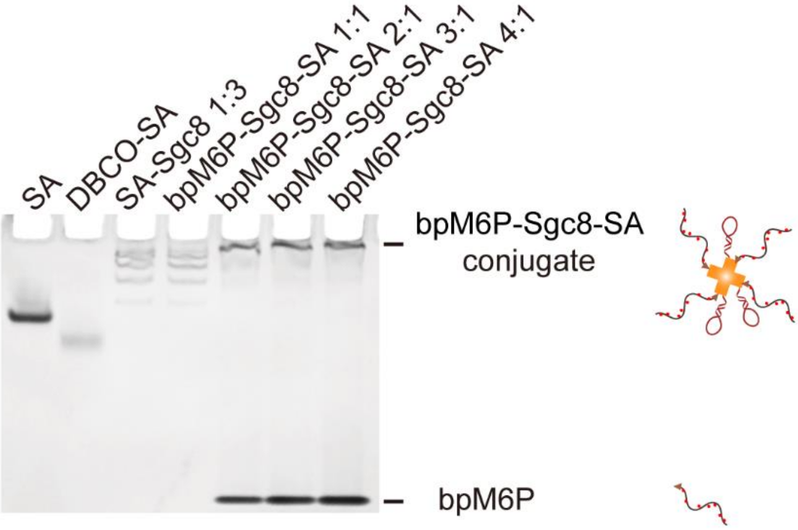

Figure S9. Native PAGE analysis of the bpM6P-Sgc8-SA conjugates with varied molar ratios under the reaction of bpM6P:SA-Sgc8 (1:1, 2:1, 3:1, and 4:1).

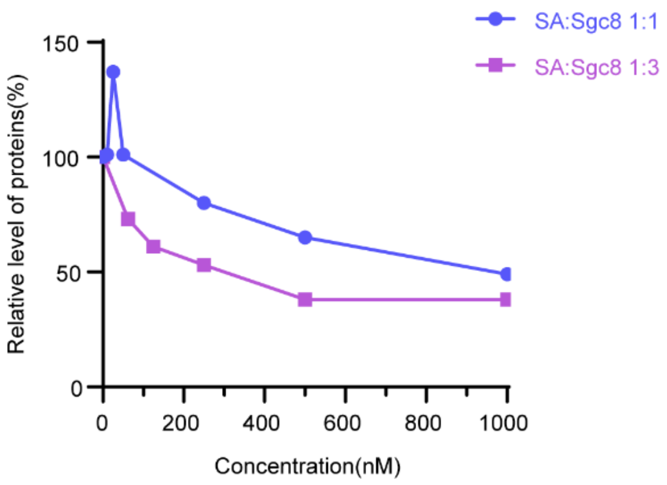

Figure S10. The ImageJ analysis of western blot (Figure 3B-C).

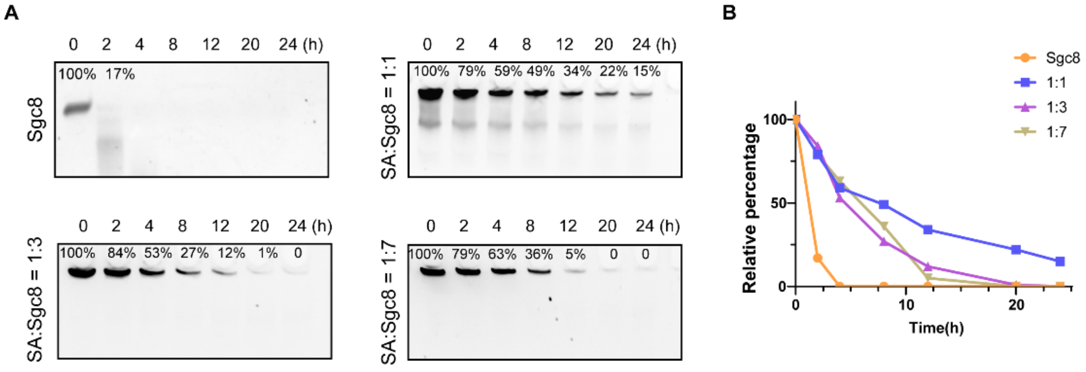

Figure S11. Stability of Sgc8 and multivalent bpM6P-Sgc8-SA (1 μM) with varied molar ratios under the reaction of SA:Sgc8 (1:1, 1:3, and 1:7) in RPMI-1640 with 20% fetal bovine serum (FBS) for different periods of time (0-24 h) at 37 °C and then assayed by denaturing (7 M urea) gel electrophoresis (D-PAGE) (A) and the gray scale analysis (B).

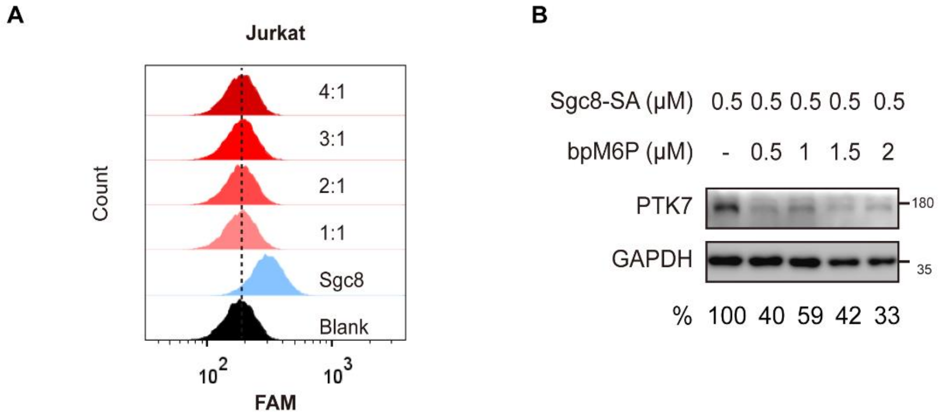

Figure S12. A) Flow cytometry analysis of bpM6P-Sgc8-SA conjugates with varied molar ratios under the reaction of bpM6P: Sgc8-SA (1:1, 2:1, 3:1, and 4:1) in Jurkat cells for 4 h. B) Western blot analysis of PTK7 expression after treatment as in (A) in Jurkat cells for 4 h.

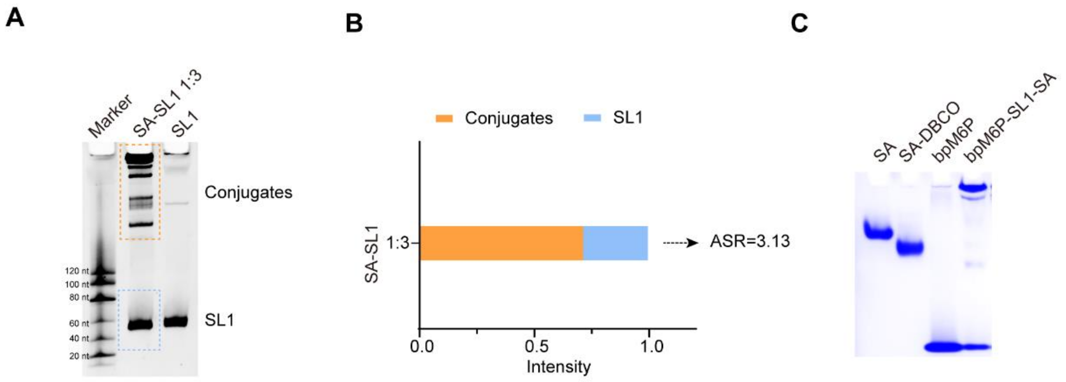

Figure S13. A) Denaturing (7 M urea) gel electrophoresis analysis of SL1-SA conjugates with varied molar ratios under the reaction of SA:SL1 = 1:3. B) ImageJ analysis of the left gel. ASR (Aptamer/SA Ratio). C) Native page analysis of bpM6P-SL1-SA conjugates (bpM6P:SL1: SA = 4:3:1).

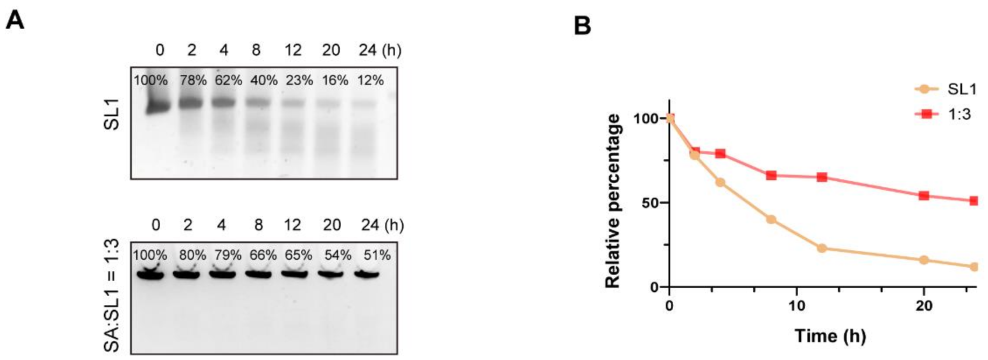

Figure S14. Stability of bpM6P-SL1-SA (SA:SL1 = 1:3, 1 μM) in RPMI-1640 with 20% fetal bovine serum (FBS) for different periods of time (0-24 h) at 37 °C and then assayed by denaturing (7 M urea) gel electrophoresis (D-PAGE) (A) and gray scale analysis (B).

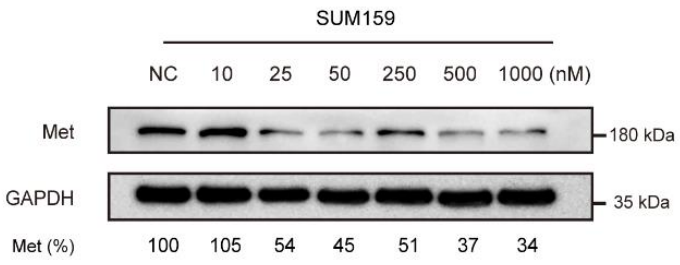

Figure S15. Western blot result of Met levels in SUM159 cells treated with multivalent AptLYTACs at different concentrations (10-1000 nM) of bpM6P-SL1-SA (SA:SL1 = 1:3) for 4 h.

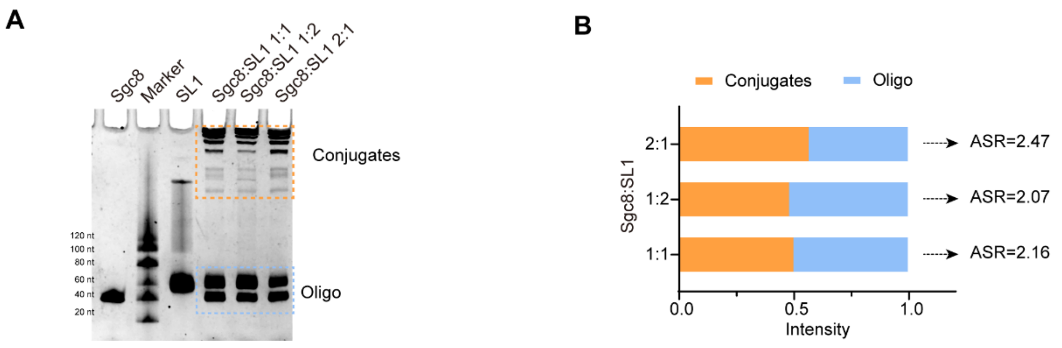

Figure S16. A) Denaturing (7 M urea) gel electrophoresis analysis of the bpM6P-Sgc8-SL1-SA conjugates with varied molar ratios (Sgc8:SL1 = 1:1, 1:2 and 2:1). B) ImageJ analysis of the left gel. ASR (Aptamer/SA Ratio)

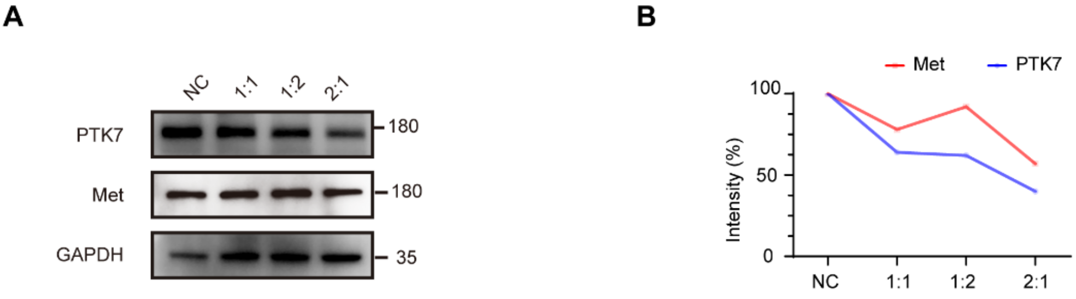

Figure S17. A) Western blot result of dual-targeted degradation by multitarget AptLYTACs (Sgc8:SL1 = 1:1, 1:2, 2:1, aptamer:SA = 3:1) after treatment without AptLYTACs (NC) or with bpM6P-Sgc8-SL1-SA (bpM6P 1 μM, Sgc8-SL1-SA 250 nM) for 4 h in SUM159 cells. B) Gray scale analysis of (A).

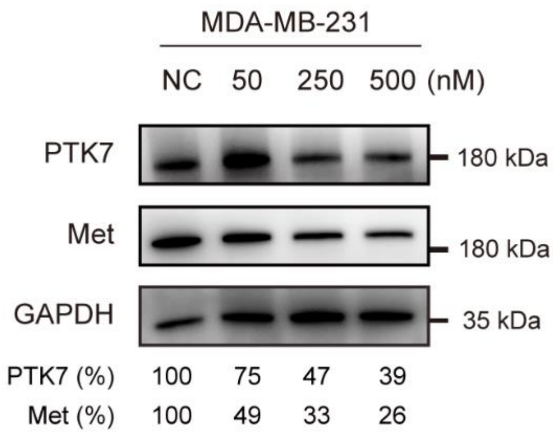

Figure S18. Western blot result showing the dual-targeted degradation efficiency by multitarget AptLYTACs (Sgc8:SL1 = 2:1, aptamer:SA = 3:1) in MDA-MB-231 cells after treatment without AptLYTACs (NC) or with bpM6P-Sgc8-SL1-SA (Sgc8-SL1-SA 50–500 nM) for 4 h.

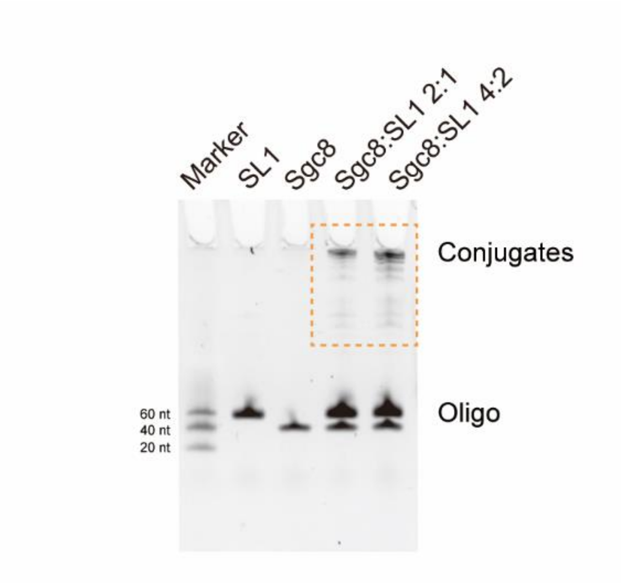

Figure S19. Denaturing (7 M urea) gel electrophoresis analysis of the bpM6P-Sgc8-SL1-SA conjugates with varied molar ratios (Sgc8:SL1:SA = 2:1:1 and 4:2:1).

Table S1. Sequences used in this work

Name	Sequence (5'-3')
Sgc8	N_3-AAAAATCTAACTGCTGCGCCGCCGGGAAAATACTGTACGGTTAGA
SL1	N_3-ATCAGGCTGGATGGTAGCTCGGTCGGGGTGGGTGGGTTGGCAAGTCTGAT
NS[a)]	N_3-ATTGCACTTACTATATTGCACTTACTATATTGCACTTACTATATTGCACTTACTATATTGCA
FAM-Sgc8	N_3-AAAAATCTAACTGCTGCGCCGCCGGGAAAATACTGTACGGTTAGA-FAM
FAM-SL1	N_3-ATCAGGCTGGATGGTAGCTCGGTCGGGGTGGGTGGGTTGGCAAGTCTGAT-FAM
FAM-NS[a)]	N_3-ATTGCACTTACTATATTGCACTTACTATATTGCACTTACTATATTGCACTTACTATATTGCA-FAM

[a)]negative sequence
